# Carbon Emissions Associated with Patient Travel for Visits at a Pediatric Tertiary Care Center: A Retrospective Analysis

**DOI:** 10.1177/22925503251411856

**Published:** 2026-01-07

**Authors:** Kayoung Heo, Chanwoo Pyo, Alice Duan, Rebecca Courtemanche, Young Ji Tuen, Douglas J. Courtemanche

**Affiliations:** 1 Department of Psychiatry, 8166 University of British Columbia, Vancouver, Canada; 2 Department of Mechanical Engineering, 8166 University of British Columbia, Vancouver, Canada; 3 Office of Pediatric Surgical Evaluation and Innovation, 37210 British Columbia Children's Hospital, Vancouver, Canada; 4 Division of Plastic Surgery, 12358 University of British Columbia, Vancouver, Canada; 5 Division of Plastic Surgery, British Columbia Children's Hospital, Vancouver, Canada

**Keywords:** Carbon emissions, healthcare, patient travel, planetary health, climate change, changements climatiques, déplacements des patients, émissions de carbone, santé de la planète, système de santé

## Abstract

**Background:** Carbon emissions associated with patient travel for hospital visits contribute to climate change. This study estimated carbon emissions from patients travelling for in-person visits and carbon emissions associated with virtual health visits at the BC Children's and Women's Hospital Campus in 2021-2022. **Methods:** Anonymized visit data categorized by in-person/virtual, and aggregated by patients’ home city/town, and fiscal quarter was obtained. Mode of travel assumptions were based on economic region and seasonality. Carbon dioxide equivalent emissions (CO_2e_) for a realistic scenario and alternative scenarios were calculated using equations derived from reference data. **Results:** There were 397,962 patient visits (19% virtual) associated with an estimated 10,001 metric tons of CO_2e_. Alternative scenarios showed that if patients from Northern or Interior regions travelled by plane during the winter, emissions decreased (−14%). While the proportion of visits that were virtual ranged from 16% to 40% by region, if all regions had 40% virtual visits, emissions would decrease by 14%. The largest reduction in carbon emissions (−26%) was found in the scenario where patients from Northern and Interior regions travelled by plane in the winter and the proportion of virtual visits increased to 40%. **Conclusion:** These findings underscore the need to raise awareness of the carbon footprint of healthcare related travel. The study urges a thoughtful consideration of planetary health when choosing between in-person and virtual visits, recognizing the ability to lower emissions by conducting virtual visits where appropriate.

## Résumé

**Introduction :** Les émissions de carbone associées aux déplacements des patients qui se rendent à un rendez-vous hospitalier contribuent aux changements climatiques. La présente étude a évalué les émissions de carbone des patients qui se déplacent en vue de rendez-vous en personne et celles reliées aux rendez-vous virtuels au campus du BC Children's and Women's Hospital en 2021–2022. **Méthodologie :** Les chercheurs ont recueilli des données anonymisées sur les rendez-vous, divisés entre les rendez-vous en personne et virtuels et regroupés selon la ville d’origine des patients et le trimestre fiscal. Ils ont présumé le mode de déplacements d’après la région économique et les facteurs saisonniers. De plus, ils ont calculé les émissions exprimées en équivalent dioxyde de carbone (éqCO_2_) pour obtenir un scénario réaliste et d’autres scénarios à l’aide d’équations dérivées des données de référence. **Résultats :** Au total, 397 962 rendez-vous de patients (dont 19% virtuels) étaient associés à une quantité estimative de 10 001 tonnes métriques d’éqCO_2_. Les autres scénarios ont révélé que si les patients des régions du Nord et de l’intérieur se déplaçaient en avion pendant l’hiver, les émissions diminuaient (−14%). La proportion de rendez-vous virtuels variait de 16% à 40% par région; si 40% des rendez-vous étaient virtuels dans toutes les régions, les émissions diminueraient de 14%. La plus grande réduction des émissions de carbone (−26%) a été observée dans le scénario où les patients des régions du Nord et de l’intérieur se déplaçaient en avion pendant l’hiver et où la proportion de rendez-vous virtuels augmentait de 40%. **Conclusion :** Ces résultats soulignent l’importance de faire connaître l’empreinte carbone des déplacements pour recevoir des soins. L’étude préconise de réfléchir à la santé de la planète au moment de choisir un rendez-vous en personne ou virtuel, puisqu’il est possible de réduire les émissions grâce à des rendez-vous virtuels lorsqu’ils conviennent.

## Introduction

Climate change has adverse effects on humans and the environment. With permanent losses to ocean and fresh-water ecosystems, as well as extreme floods, droughts, and storms, humans have experienced more food and water insecurity, diseases, mental health challenges, trauma, and loss of income and culture.^1^ People living in highly vulnerable regions experience these effects 15 times higher compared to those in low vulnerable regions.^1^ Climate change is partially caused by anthropogenic greenhouse gas (GHG) emissions, which can be categorized as scope 1 (direct), scope 2 (indirect from energy consumption), and scope 3 (indirect from buying, using, and disposing products).^2^ Scope 3 typically make up the majority of a company's carbon footprint, and have been a focus of decarbonization efforts.^2^ The mortality impact of carbon dioxide has been estimated by Bressler, who found that the lifetime emissions of 3.5 average Americans (4434 metric tons of CO_2_) causes one excess death.^3^ However, Bressler also quantified the direct benefit of decarbonization where if the carbon dioxide equivalent (CO_2e_) emissions from a coal powerplant were removed, over 900 lives would be saved.^3^ Ironically, healthcare contributes to climate change, with Canada's healthcare system accounting for 4.6% of the country's total GHG emissions,^4^ with 50%–75% of these emissions considered indirect (scope 3).^5^

An urgent need to lower carbon emissions has led to greater awareness, leadership, and research around sustainable healthcare.^6^ Medical and physician colleges have divested from fossil fuels, acknowledged the unintended negative impact of healthcare on the public, and declared climate change a medical and population health emergency.^7^ Within Canada, a group of health professionals (Canadian Association of Physicians for the Environment, CAPE) work to engage government and draw media attention to key issues.^8^ Clinical and environmental researchers have shown the environmental benefits of newer hospital buildings, using reusables over disposables, and stopping the use of anesthetic gases with high global warming potential.^9–12^

Recent published reports have quantified CO_2e_ emissions associated with patient travel and CO_2e_ savings from using telemedicine/virtual visits.^13,14^ In England, 4.9% of CO_2e_ emissions in 2019 produced by the NHS were associated with patient travel (123 Kt of CO_2e_).^15^ Other reports have focused on specific patient groups. Yusuf et al found 8235 roundtrip patient visits to the hospital in one year for cancer care resulted in 32 metric tons of CO_2e_.^14^ In an Australian dialysis unit, patient travel accounted for 7 metric tonnes of CO_2e_ (5.8% of their total GHG emissions in a year).^16^ The potential CO_2e_ emission savings from switching to telemedicine/virtual visits were shown by Patel et al, where over the course of 15 months, nearly 50,000 in-person visits were shifted to telemedicine visits for patients with cancer living in Florida, resulting in a savings of 6989 metric tons of CO_2e_.^13^ Although these reports highlight the significance of carbon emissions associated with hospital travel, these findings are not directly transferrable to other healthcare regions and patient populations.

In British Columbia (BC), Canada, the BC Children’s and Women’s Hospital (C&W) Campus includes the province's only pediatric care center as well as a pediatric mental health center and a women's tertiary care center. In 2021, BC's population was just over five million, of which 19% were 0-19 years old.^17^ BC is geographically diverse; about 75% of the population can travel to C&W in 3-4 h via car and/or ferry, while the remaining 25% who live in Northern or Interior regions must drive for a full day or take a flight to get to the hospital.^18^ To date, no studies have looked at CO_2e_ emissions associated with patient travel to this hospital center. Therefore, we aimed to estimate CO_2e_ emissions associated with patient visits from April 1, 2021 to March 31, 2022. Our primary objective was to estimate CO_2e_ emissions for all visits based on a realistic scenario considering visit type (in-person/virtual) and mode of travel assumptions (driving/flying/ferrying). Our secondary objectives were to estimate CO_2e_ emissions based on three alternative scenarios: (1) a higher proportion of patients flying in the winter, (2) a higher proportion of patients driving in the winter, and (3) a higher proportion of virtual visits.

## Methods

This study was a retrospective review of administrative data. Ethics approval was obtained from the University of British Columbia C&W Clinical Research Ethics Board (H23-00541).

### Patient Visits

All patient visits at the C&W Campus for fiscal year 2021/22 were obtained from the hospital database. The C&W Campus includes BC Children’s Hospital, BC Mental Health & Substance Use Services and BC Women’s Hospital and Health Centre. Visits were aggregated by city/town of residence, fiscal quarter, and categorized by visit type (in-person or virtual). For city/towns with fewer than five appointments, the city/town was categorized as ‘unidentified’.

### Mode of Travel Assumptions

**Figure 1.**
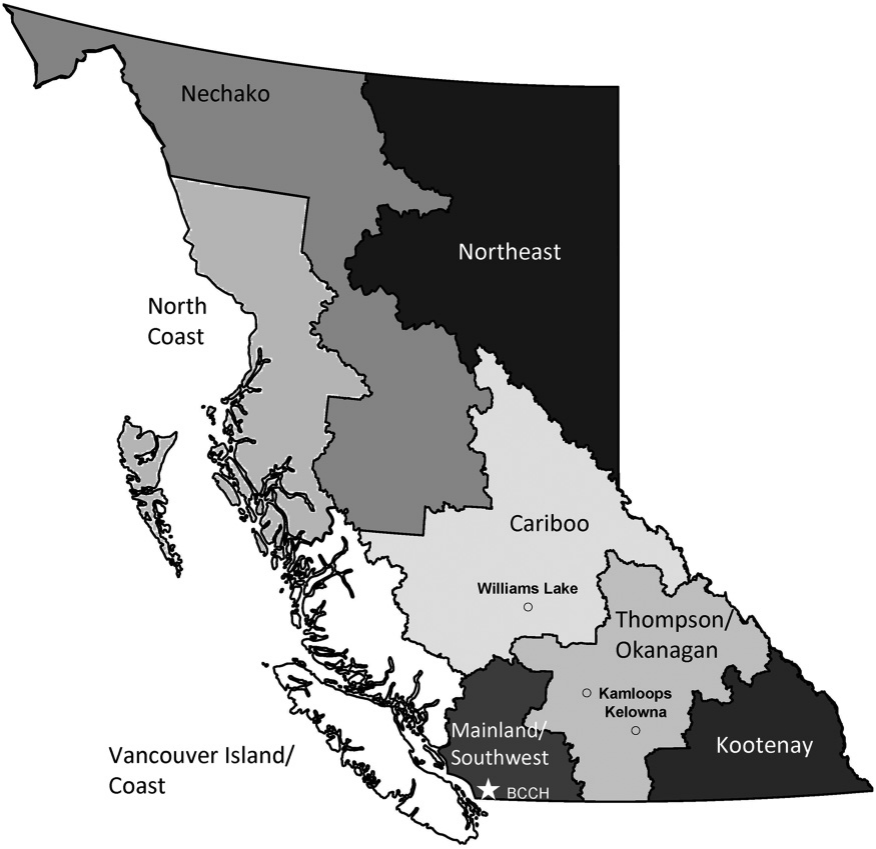
BC Regions with BC Children’s Hospital (BCCH), Kelowna, Kamloops, and Williams Lake Shown for Reference. Map Created in R Statistical Software Version 2024.12.1 + 563 Using the Package ‘Bcmaps’ Version 2.2.1^20^ and Adobe Acrobat Pro.

To make assumptions about how patients travelled to the hospital for in-person visits (ie, driving, flying, ferrying), a realistic scenario based on regional geography and seasonality was considered. Economic regions were chosen over Health Authority regions as there are more economic regions, and thus the resultant CO_2e_ emission data would be more geographically informative.^19^ The realistic scenario assumed that patients from Northern and Interior regions of the province would drive more during summer months and fly more during winter months, and patients traveling from outside of BC would fly, Figure 1, Table 1.

**Table 1.** Realistic Scenario.

BC Region(s)	April – September		October – March	
	*Drive*	*Fly*	*Drive*	*Fly*
Northern (Nechako, Cariboo, Northeast, North Coast) ^a^	90%	10%	50%	50%
Interior (Kootenay, Thompson-Okanagan) ^b^	90%	10%	50%	50%
Vancouver Island/Coast ^c^	100%	0%	100%	0%
Mainland/Southwest	100%	0%	100%	0%
Out of Province ^d^	0%	100%	0%	100%

^a^ Families who live in Williams Lake or further from BCCH will drive 90% of the time from April to September, and 50% of the time from October to March.

^b^ Families who live in Kamloops or Kelowna or further from BCCH will drive 90% of the time from April to September, and 50% of the time from October to March.

^c^ Families who live on Vancouver Island will drive and take the ferry 100% of the year. Because the ferry ride from the Sunshine Cost to Vancouver is a short trip (∼45 min), the ferry ride was considered driving distance for the purposes of estimating CO_2e_ emissions.

^d^ Families who live out of province were assumed to travel via major airports in equal proportions (eg, for patients residing in Alberta, 50% of patients were assumed to travel via Calgary airport and 50% via Edmonton airport).

Alternative scenarios were also considered to explore how carbon emissions would be affected by mode of travel during winter and increasing the proportion of virtual visits to 40%. A 60:40 ratio of in-person to virtual visits was chosen based on our finding that the highest proportion of virtual visits by region was 40% (Kootenay), suggesting this 60:40 ratio may be feasible in all geographic regions.

1. Winter flying scenario: 100% of families living in the Northern region (North or East of Williams Lake) and Interior regions (North or East of Kamloops or Kelowna) would fly from October to March, Figure 1.
2. Winter driving scenario: 100% of families would drive from October to March.
3. Virtual visit scenario: 40% of all visits were virtual.

Carbon emission estimates based on mode of travel were calculated according to the equations listed in Table 2. The following paragraphs describe how each equation was derived and what assumptions were made.

**Table 2.** CO_2e_ Emission Equations.

Equation	Mode of Travel	Equation (metric tons per visit)
1	Driving	*Distance Driven_ km_ x 259 _g/km_ x 2 _ways_ x 1* *×* *10^−6^_metric tons/g_*
2	Ferrying	*0.11286 _kg CO_*_2e*/km*_ *x (ferry route distance _km_) x 2 people x 2 ways x 0.001 _metric ton/kg_*
3	Flying	*CO*_2e_ *Produced from Flying Round Trip x 2 _people_*
4	Virtual Visit	*45.5 g _CO2e/GB_ x 1.4 _GB/1hr_ x 1 h_/visit_ x 1* *×* *10^−6^_metric tons/g_* *=* *0.000637 metric tons CO2/visit (63.7 g CO2e/visit)*

### Carbon Emission Estimates – Driving

CO_2e_ emissions from driving were calculated using distance in kilometers between patients’ geographic home to C&W using Google Maps.^21^ An estimate of the average CO_2e_ emission per kilometer driven was calculated based on published Statistics Canada vehicle data for BC and CO_2e_ emission fuel type data. Using Statistics Canada BC Vehicle registration data, vehicles were categorized by fuel type: gasoline, diesel, plug-in hybrid, and battery electric, Table 3.^22^ The average CO_2e_ emission (g/km) for each fuel type was found using the Canadian Fuel Consumption Guide 2022, which lists vehicles by brands, models, and fuel types.^23^ By applying the proportions of vehicles driven by BC residents, the average CO_2e_ emission per vehicle was found to be 259 g/km. Using this information, equation 1 (Table 2) was derived to calculate the total CO_2e_ emission produced from a patient's round-trip travel from home to C&W.

**Table 3.** Vehicle by Fuel Type in the Province of British Columbia.^22^ Vehicles weighing < 4535 kg included.

	Gasoline	Diesel	Battery electric	(Plug-in) Hybrid electric	Other fuel types^a^	All fuel types
Vehicle Count	2,950,136	124,739	48,056	86,066	196	3,209,193
Vehicle Proportion	91.93%	3.89%	1.50%	2.68%	0.01%	100%
CO_2e_ Emissions (g/km), average	267.803	272	0	99.4	−	−
CO_2e_ Emission (g/km) per vehicle, weighted average	259					

^a^ Other: liquid propane, natural gas, hydrogen, etcetera.

### Carbon Emission Estimates – Ferrying

The three major BC ferry routes were considered to estimate CO_2e_ emissions: Swartz Bay to Tsawwassen (Route 1), Horseshoe Bay to Nanaimo (Route 2), and Tsawwassen to Nanaimo (Route 3), Table 4. The remaining ferry routes, many of which are less frequently used, or are much shorter (less than one hour crossing time) was considered to be driving distances for the purposes of the study.

**Table 4.** BC Ferries Major Routes by Distance and Crossing Time.

Route	Distance (km)	Crossing Time
Route 1: Swartz Bay to Tsawwassen	44.45 km	1hr 35min
Route 2: Horseshoe Bay to Nanaimo	59.26 km	1hr 35min
Route 3: Tsawwassen to Duke Point	68.52 km	2hr

BC Ferries does not publish CO_2e_ emissions by passenger type and route. Therefore, a reference value for the average CO_2e_ emission per passenger kilometer (0.11286 kg CO_2_/km) based on all passenger types (foot/car) was obtained from the European Union certified travel sustainability platform, Thrust Carbon.^24^ This CO_2e_ emission data was used to derive equation 2, Table 2.

The driving distances from a patient's home to the ferry terminal, and the ferry terminal to C&W were also considered to calculate CO_2e_ emissions per visit. Distances in kilometers were determined from Google Maps,^21^ and Equation 1 was applied.

### Carbon Emission Estimates – Flying

For each geographic region and time of year that an assumption was made for travel by air, it was further assumed that patients would travel from the closest airport to their home to Vancouver International Airport (YVR). Using an online carbon footprint calculator developed by Carbon Footprint Ltd, a UK registered company,^25^ the carbon footprint for a roundtrip flight to YVR for each BC airport was found, Table 5. The amount of CO_2e_ emissions produced from flights was calculated using Equation 3, Table 2.

**Table 5.** CO_2e_ Emissions from BC Airports for a Round-Trip Flight to Vancouver International Airport (YVR).

BC Airport	CO_2e_ emissions (metric tons)
YDL: Dease Lake	0.17
YYE: Fort Nelson	0.16
ZMT: Masset	0.12
YXJ: Fort St. John	0.12
YZP: Sandspit	0.11
YPR: Prince Rupert	0.11
YDQ: Dawson Creek	0.11
YXT: Terrace	0.10
YYD: Smithers	0.10
ZST: Stewart	0.09
TUX: Tumbler Ridge	0.09
CYCQ: Chetwynd	0.09
YPZ: Burns Lake	0.09
YRV: Revelstoke	0.09
CZAM: Salmon Arm	0.09
CYVK: Vernon Regional	0.09
YXC: Cranbrook/Canadian Rockies	0.09
YXS: Prince George	0.08
ZEL: Bella Bella	0.07
QBC: Bella Coola	0.06
YAA: Anahim Lake	0.06
YCG: Castlegar	0.06
YQZ: Quesnel	0.06
YWL: Williams Lake	0.05
YZT: Port Hardy	0.05
YYF: Penticton Regional	0.04
YKA: Kamloops	0.04
YLW: Kelowna International	0.04

The driving distances from a patient's home to their nearest airport, and YVR to C&W were also considered to calculate CO_2e_ emissions per visit. Distances in kilometers were determined from Google Maps,^21^ and Equation 1 was applied, Table 2.

### Carbon Emission Estimates – Virtual Visits

CO_2e_ emissions for virtual health visits were estimated using available data on CO_2e_ emissions from Internet use, and average Internet use for virtual health visits. A study by Obringer et al in 2021 found CO_2e_ emissions range from 28 to 63 grams per gigabyte of Internet (average 45.5 g).^26^ According to the online software platform Coreplus, which manages telehealth services for health professionals, a one-hour virtual visit at 720p resolution, requires an average of 1.2 GB to 1.4 GB of Internet.^27^ Although the length of virtual visits varies by specialty type and visit type, for the purposes of this study, it was assumed virtual health visits would take one hour. Thus, the total CO_2e_ emission for a virtual visit was calculated according to equation 4, Table 5.

### Data Analysis

Hospital visits were tabulated by patient home region and visit modality (in-person/virtual), visualized on a map of BC. Carbon emissions were calculated according to study equations and mode of travel assumptions for the realistic scenario. CO_2e_ emissions by region were estimated and visualized on a map of BC. Additionally, using population estimates from the government of BC,^19^ per person carbon emissions by regional population were estimated and visualized on a map of BC. For each alternative scenario, carbon emissions were estimated and compared with carbon emissions from the realistic scenario.

## Results

### Patient Visits

**Figure 2.**
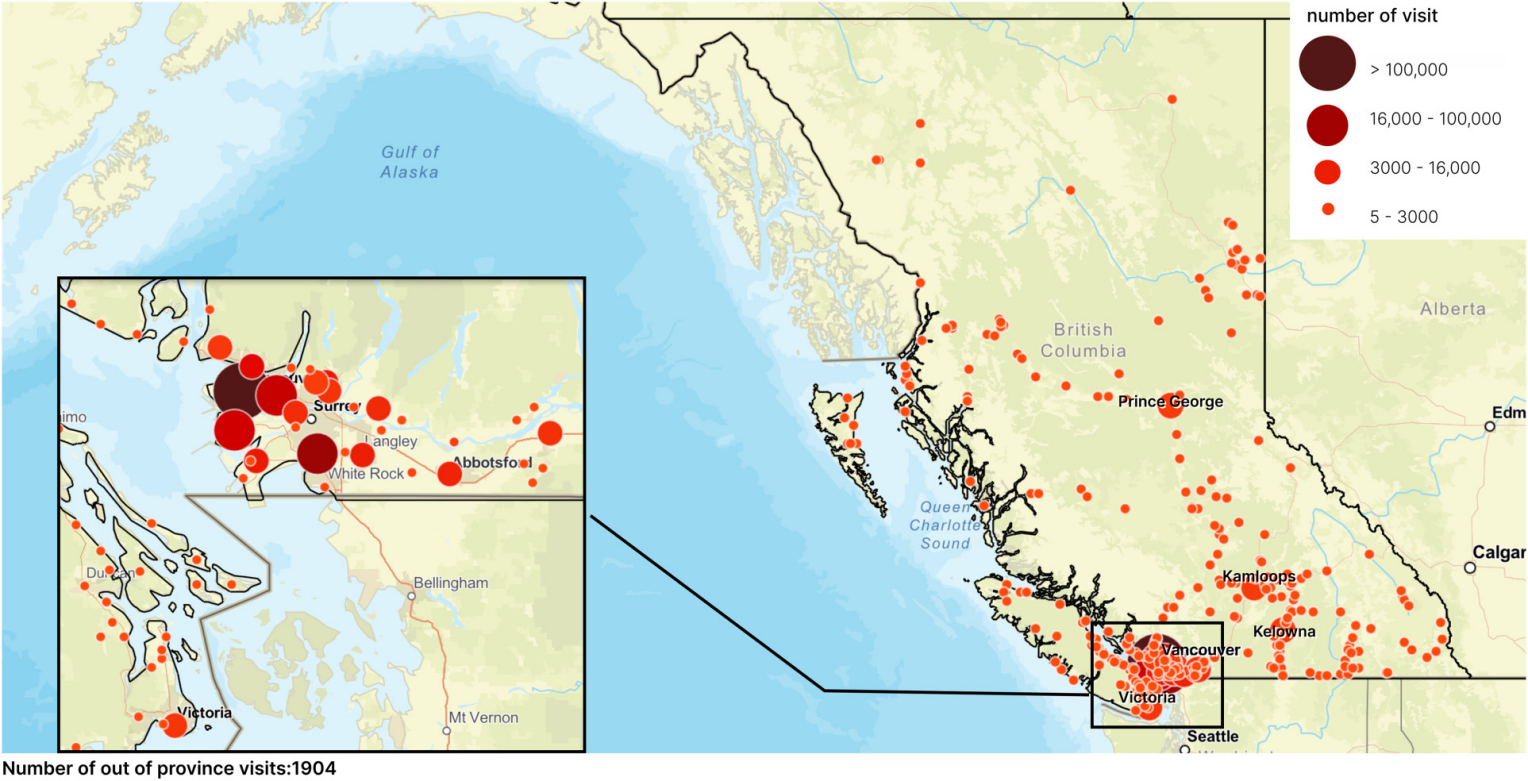
Number of Visits by Patients’ Home City/Town.

A total of 397,962 visits occurred during the fiscal year 2021/22. The majority of visits were for patients living in the Mainland/Southwest region (87%), followed by the Vancouver Island/Coast region (4%), Figure 1, Figure 2, Table 6. Of the 397,962 total visits, 19% were virtual. By region, patients living in the Mainland/Southwest region had the highest percentage of in-person versus virtual visits (84% vs 16%), while patients living in the Vancouver Island/Coast or Kootenay regions had the lowest percentage of in-person versus virtual visits, (61% vs 39%) and (60% vs 40%), respectively.

**Table 6.** Visits by Patient Home Region and Modality.

BC Region	Number of Visits	Modality	
		In-person	Virtual
Mainland/Southwest	345727 (87%)	84%	16%
Vancouver Island/Coast	16272 (4%)	61%	39%
Thompson-Okanagan	15414 (4%)	63%	37%
Cariboo	5717 (1%)	65%	35%
North Coast	3294 (1%)	72%	28%
Kootenay	2826 (1%)	60%	40%
Out of Province	2737 (1%)	76%	24%
Northeast	2443 (1%)	67%	33%
Nechako	1830 (0.5%)	70%	30%
Unknown	1702 (0.4%)	73%	27%
**All Regions**	**397962** (**100%)**	**81%**	**19%**

### Carbon Emission Estimates – Realistic Scenario

**Figure 3.**
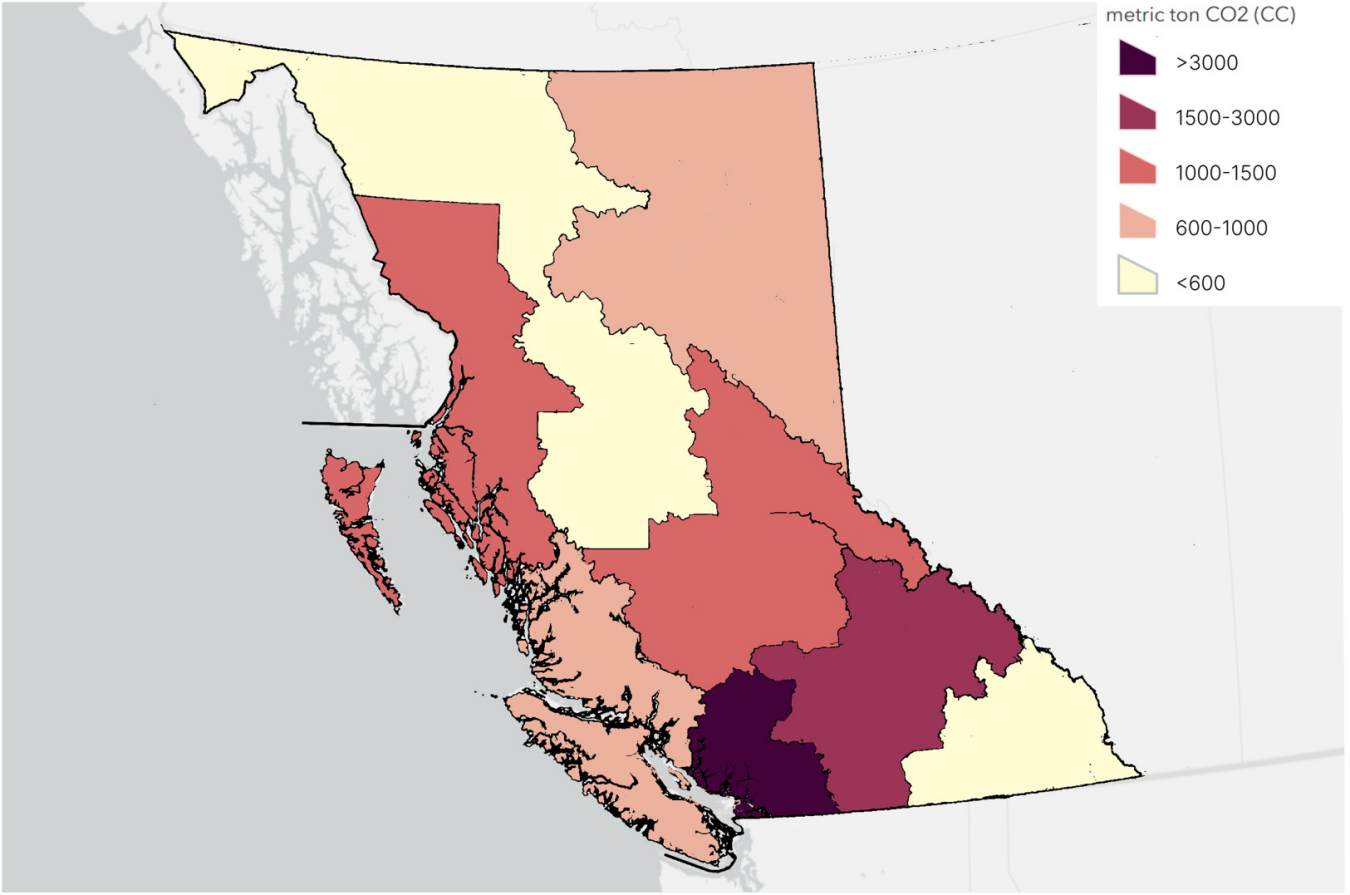
CO_2e_ Emissions by Region. Out-of-Province Visits Accounted for 11 Metric Tons.

In the realistic scenario, in-person patient visits (81%) collectively contributed to an estimate of 10,001 metric tons of CO_2e_ emissions. Virtual visits, which accounted for 19% of all visits, contributed an estimate of 4.8 metric tons of CO_2e_ emissions (0.05% of the total CO_2e_ emissions from all visit types). The Mainland/Southwest region, which accounted for the highest proportion of visits (87%) contributed the largest amount of CO_2e_ emissions (>3000 metric tons of CO_2e_), while the Nechako region (least populated region of the province) and the Kootenay region contributed the least (<600 metric tons of CO_2e_), Figure 3.

**Figure 4.**
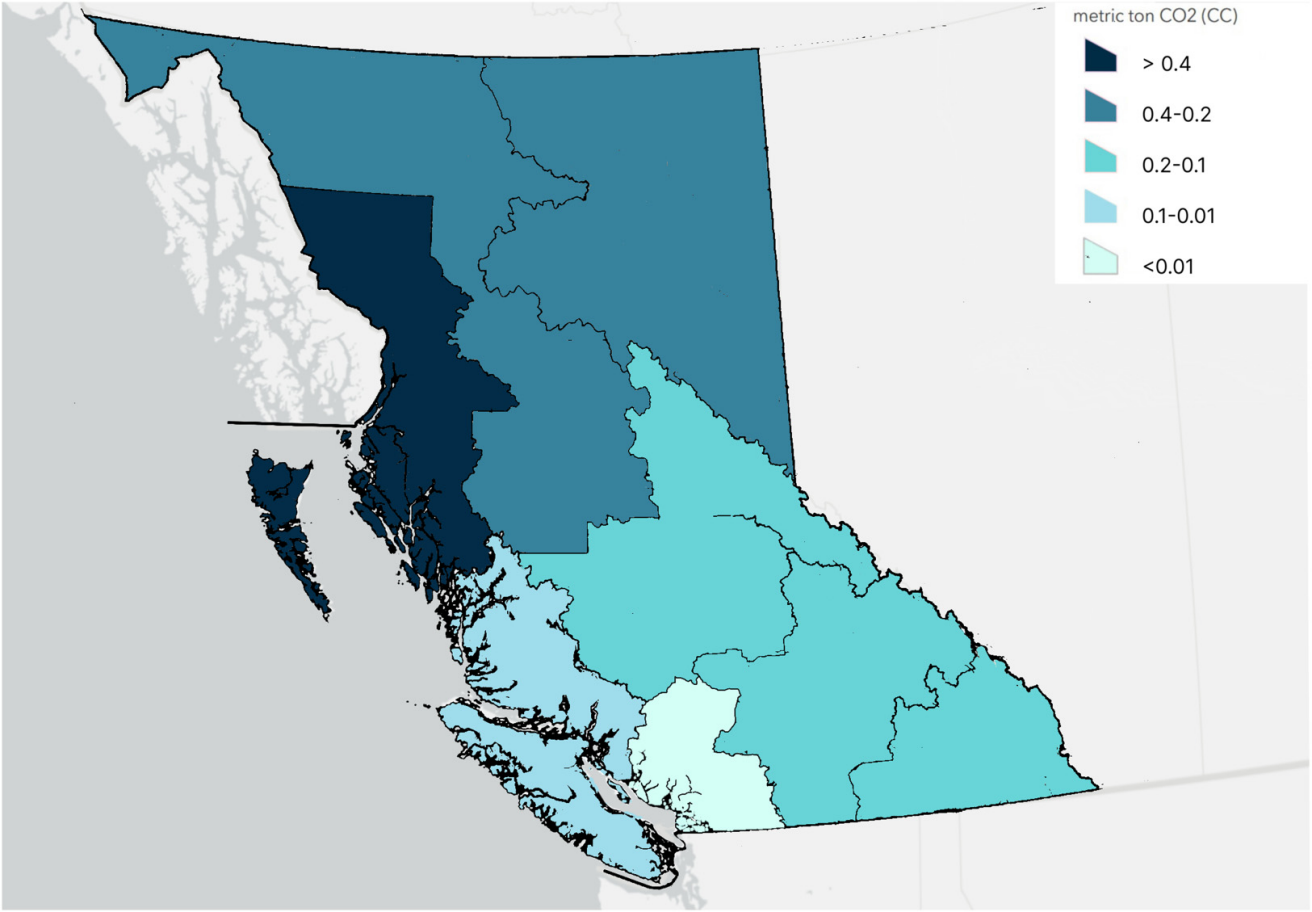
CO_2e_ Emissions for Visits Normalized by Regional Population.

When considering CO_2e_ emissions per visit by regional populations, the North Coast had the highest CO_2e_ emissions per person (>0.4 metric tons), whereas the Mainland/Southwest had the least CO_2e_ emissions by per person (<0.01 metric tons), Figure 4.

### Carbon Emission Estimates – Alternative Scenarios

Compared to carbon emissions from in-person visits estimated in the realistic scenario, the winter flying scenario would reduce CO_2e_ emissions by 14% to 8618 metric tons, Table 7. In the winter driving scenario, CO_2e_ emissions would increase by 14% to 11,384 metric tons. In the final scenario (if 40% of all visits were virtual) 8602 metric tons of CO_2e_ emissions would be produced, representing a 14% reduction in CO_2e_ emissions compared to the realistic scenario. Putting this into perspective, if a patient living in Kelowna drove to an in-person appointment, 202 kg of CO_2e_ would be produced, whereas only 0.06 kg of CO_2e_ would be produced from a one-hour virtual visit. Realistically, not all hospital visits can be virtual. However, increasing the proportional of all visits that could be virtual from 19% to 40%, would save 1398 metric tons of CO_2e_, which is the equivalent to saving greenhouse gas emissions from 326 gasoline-powered passenger vehicles driven for one year.^28^ Combining the effects of increasing virtual visits to 40% and increasing the proportion of patients who fly during winter would result in a savings of 7358 metric tons of CO_2e_ (26% less CO_2e_ emissions than realistic scenario).

**Table 7.** CO_2e_ Emission Estimates.

Scenario	CO_2e_ emissions (metric tons)	% Change in CO_2e_ emissions from realistic scenario
Realistic	10,001	n/a
Winter driving	11,384	+14%
Winter flying	8618	−14%
Virtual visits (40%)	8602	−14%
Virtual visits (40%) + Winter driving	9847	−2%
Virtual visits (40%) + Winter flying	7358	−26%

## Discussion

This study found one year of patient travel to the C&W campus resulted in an estimated 10,001 metric tons of CO_2e_ emissions. Emissions were found to reduce in three alternative scenarios. If patients from Northern and Interior regions traveled by plane instead of car/truck during the winter season (October-March), 1383 metric tons of CO_2e_ emissions would be saved. Moreover, increasing the proportion of virtual visits from 19% to 40%, would reduce carbon emissions by 1399 metric tons. Finally, the combination of individuals living in the Northern and Eastern regions flying in winter, and increasing the proportion of virtual visits resulted in a 26% reduction in CO_2e_, a savings of 2643 metric tons of CO_2e_ – the equivalent to greenhouse gas emissions from 616 gasoline-powered passenger vehicles for one year.^28^

Compared to other published reports, this study estimated emissions from patient travel at a provincial healthcare site rather than focusing on a particular specialty type (eg, patient travel to oncology appointments) or describing emissions from a national healthcare system.^13–15^ Despite differences in study populations/regions, our results corroborates other reports that showed decreases in CO_2e_ emissions when in-person appointments were made virtual.^13^ This study differs from previous reports in that we explored a hypothetical scenario of how a higher proportion of virtual visits (up to 40% of all visits made virtual) would change the amount of CO_2e_ saving. Although we expected to find a larger decrease in carbon emission by increasing virtual visits from 19% to 40%, this finding is likely explained by the fact that there is already a high proportion of virtual visits (24% - 40%) in all regions except Mainland/Southwest BC. Nonetheless, reducing emissions by increasing the proportion of virtual visits to 40% would still result in significant carbon savings (1398 metric tons of CO_2e_). This suggests that in the context of climate change, virtual visits should be considered where possible, even for patients living close to the hospital.

Carbon emissions were also found to decrease in the winter flying scenario compared to the realistic scenario. Although other reports have not explored seasonality, the relevance of this variable depends on geography. In BC, seasonality is significant for nearly all patients in the province, but more so for those travelling from Northern and Interior regions. For patients who can choose to travel by car or plane, weather conditions in the winter months may play a role in their decision making.

This study was limited by not knowing exactly how each patient travelled for their appointments and not being able to account for patients who have multiple appointments over a few days. We did not include patient travel by walking, biking, or public transit in our scenarios. We assumed that patients would be accompanied by one other adult, but we know from experience this is not always the case. Additionally, during the summer months, it's likely families will plan vacation/holiday time around the need to travel to the hospital. We were unable to subgroup our analysis by visit type (ie, inpatient, outpatient, emergency) due to data limitations; however, it's important to note that inpatient and emergency visits cannot be done virtually, and likely the majority of emergency visits were associated with patients living within the local health authority. We were also not able to distinguish virtual visits that were made via photo-phone versus via video-call. Moreover, for the purposes of the study we assumed all virtual visits would take one hour. However, this is likely an overestimate as the length of visit will depend on a specialty type and the nature of the visit (ie, initial consult vs. follow-up). Moreover, the proportion of virtual visits may be higher than current practice patterns as the time period of visits included in this study overlapped with the COVID-19 pandemic when there were public health restrictions.^29^ We were also not able to estimate carbon emission savings from outreach care in remote and rural communities. Finally, local emission data for ferry travel was not available, so we used international reference data that may not be equivalent. Although we aimed to make reasonable assumptions to estimate carbon emissions, our CO_2e_ emissions estimates may be inaccurate, but nonetheless they may help illustrates overall trends. Given these limitations, this study can be considered as a thought experiment that shows the role of mode of travel, visit type, geography, and seasonality on carbon emissions.

## Conclusion

In summary, increasing the proportion of patients who travel by plane versus car, as well as increasing the proportion of virtual visits where possible will reduce carbon emissions associated with patient travel for visits at the C&W Campus. These results may be generalizable to other provinces and territories in Canada where a single center serves a large geographic region. In the race against time with climate change, we hope the findings from our study can be adopted into practice by clinicians according to their workflow preferences and patient populations. We hope these changes, along with other efforts, will help minimize healthcare related carbon emissions and will result in noticeable climate benefits in the decades ahead. Future work should focus on other practical and effective strategies to reduce carbon emissions associated with healthcare.
